# Wanted: unique names for unique atom positions. PDB-wide analysis of diastereotopic atom names of small molecules containing diphosphate

**DOI:** 10.1186/1471-2105-9-S9-S16

**Published:** 2008-08-12

**Authors:** Christopher A Bottoms, Dong Xu

**Affiliations:** 1 Department of Computer Science and Christopher S. Bond Life Sciences Center, University of Missouri, Columbia, MO 65211, USA

## Abstract

**Background:**

Biological chemistry is very stereospecific. Nonetheless, the diastereotopic oxygen atoms of diphosphate-containing molecules in the Protein Data Bank (PDB) are often given names that do not uniquely distinguish them from each other due to the lack of standardization. This issue has largely not been addressed by the protein structure community.

**Results:**

Of 472 diastereotopic atom pairs studied from the PDB, 118 were found to have names that are not uniquely assigned. Among the molecules identified with these inconsistencies were many cofactors of enzymatic processes such as mononucleotides (e.g. ADP, ATP, GTP), dinucleotide cofactors (e.g. FAD, NAD), and coenzyme A. There were no overall trends in naming conventions, though ligand-specific trends were prominent.

**Conclusion:**

The lack of standardized naming conventions for diastereotopic atoms of small molecules has left the *ad hoc *names assigned to many of these atoms non-unique, which may create problems in data-mining of the PDB. We suggest a naming convention to resolve this issue. The in-house software used in this study is available upon request.

A version of the software used for the analyses described in this paper is available at our web site: .

## Background

Often accompanying the macromolecules deposited in the Protein Data Bank (PDB) [1] are smaller molecules of biological importance. Some of these are energy-carrying cofactors, such as ATP, coenzyme A, and nicotinamide-adenine dinucleotide (NAD). Some analogs of these molecules are either drugs or can be used in drug design [2,3].

**Figure 1:**
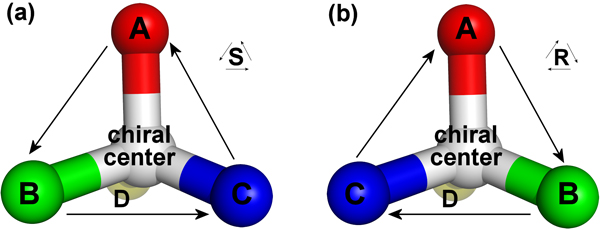
**S and R configurations for chiral centers**. (a) S configuration and (b) R configuration, for atoms A, B, C, and D when they have the highest, second, third, and lowest priorities, respectively. Notice that when the three highest priority groups (A, B, and C) are facing the viewer, they have a counter-clockwise arrangement in the S configuration and a clockwise arrangement in the R configuration.

**Figure 2:**
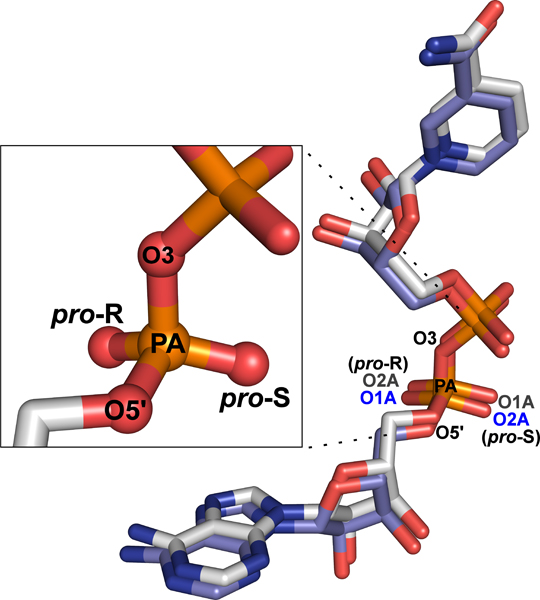
**NAD molecules from X-ray crystal structure **2OHX. For comparison purposes, one molecule is superimposed on the other and then offset slightly. The atom names are similarly offset for the diastereotopic oxygen atoms of the adenine-side phosphate group. Note the differences in names for the *pro*-R and *pro*-S atoms. Both molecules of NAD shown are from an alcohol dehydrogenase structure [PDB:2OHX][16]. Following the CIP-algorithm, since all four oxygen atoms have the same atomic number, their priority is determined by subsequent bonded groups. The O3 oxygen atom is bonded to the next phosphorus atom and the O5' oxygen atom is bonded to the preceding C5' carbon atom, while the remaining two oxygen atoms are unbonded except to the original phosphorus atom. Therefore, the O3 oxygen atom has the highest priority, the O5' oxygen atom has the second highest priority, and the remaining two oxygen atoms tie for the lowest priority. The *pro*-S atom is the one that, if it were replaced with an atom of highest priority, would make the phosphorus atom chiral with an S configuration. Both molecules are drawn using red for oxygen, blue for nitrogen, and orange for phosphorus. One is drawn using light blue for carbon and the other is drawn using white for carbon.

Like other biologically relevant molecules, many of these small molecules contain chiral or prochiral centers. An atom is a chiral center if four different chemical groups are attached to it. A chiral configuration can be designated R or S, depending on the arrangement of the attached groups (Figure 1). If, however, two of these groups are identical, then the center atom is prochiral, meaning that it would become chiral if either of the identical groups were substituted for a unique group. These two groups are called diastereotopic, i.e., if either were replaced with a unique group, the molecule would become one or another diastereomer. Within a pair of diastereotopic atoms, one is designated *pro*-R and the other *pro*-S, indicating the configuration of the chiral atom would result from replacing the diastereotopic atom with a group that has higher priority than the other groups. Many ligands contain diphosphate groups that contain at least one prochiral phosphorus atom (Figure 2).

The *pro*-S and *pro*-R oxygen atoms of nucleic acid strands are named "OP1" and "OP2", respectively [4]. Many enzymes treat the *pro*-R and *pro*-S oxygen atoms of DNA and RNA differently [5]. These diastereotopic oxygen atoms are also treated differently in RNA-intron splicing [6,7]. Small diphosphate-containing molecules also participate in enzymatic reactions in which the distinction between diastereotopic atoms or groups is important [5,8,9]. Unfortunately, many of these diastereotopic atoms do not have standardized names, an issue that has not been investigated to our knowledge. Consistent naming of diastereotopic atoms is needful when performing all-atom superpositioning or all-atom root mean square deviation (RMSD) calculation [10]. It is also needful for data mining in the PDB, e.g., structure-based virtual screening for drug candidates [11,12]. In this paper, we will conduct a systematic PDB-wide analysis on the diastereotopic atom names of small molecules containing diphosphate.

## Results

### Inconsistencies in PDB files

There were 4167 PDB files containing a total of 295 distinct ligands having prochiral centers that met our strict criteria. Over half of these ligands (175) had two prochiral phosphate centers that were adjacent to carbon, and one had three (OXT from [PDB:2JI7] [13]), for a total of 472 distinct prochiral centers adjacent to carbon. For example, NAD contains two because it has a diphosphate sandwiched between two ribose moieties. Each distinct prochiral center contains a pair of disastereotopic atoms. We analyzed the names of the atoms at each prochiral center. Of these distinct centers, 354 had a single naming convention but 241 of these also only occurred in a single PDB file. There were 118 distinct prochirality centers that had more than one naming convention.

We defined a case of swapped names to occur when all of the following were true between two molecules with the same type of prochiral center: (1) the highest and second highest priority names were consistent, (2) the *pro*-R atom of one prochiral center had the same name as the *pro*-S atom of a second center, and (3) the *pro*-S atom of the first center had the same name as the *pro*-R atom of the second center (Figure 2). 117 of the 118 centers had swapped naming conventions as defined above. The remaining center, which had two naming conventions, actually had a naming error. Nine of the 117 centers with swapped names had additional naming conventions. In every case, we found that the extra naming conventions were caused by errors rather than mere inconsistencies. For example, in a structure of a surfactin synthetase-activating enzyme [PDB:1QR0] [14], the diastereotopic atoms attached to phosphorus atom P1A are labeled "O5A" and "O4A" instead of the names "O2A" and "O1A" defined in the Chemical Component Dictionary  from the PDB. In a similar manner, the diastereotopic atoms attached to P2A are named "O2A" and "O1A", instead of the names "O5A" and "O4A" defined in the dictionary file. In another example, in a structure of *E. coli *carbamoyl phosphate synthetase [PDB:1CE8] [15] the O5' oxygen atom is mislabeled as O4' for 8 different ADP molecules. Interestingly, in four of these molecules, the *pro*-S and *pro*-R atoms are labeled "O1A" and "O2A", respectively, while in the other four molecules they are labeled "O2A" and "O1A", respectively.

In Table 1, we present statistics for sample cases in which there were at least two nonredundant examples of each naming convention. For additional selected examples, see Supplement Table 1 in Additional File 1. For our full results, including cases that had no inconsistencies, see Supplemental Table 2 in Additional File 2 (explanation in Additional file 3). All results, including those resulting from errors, are included in Supplemental Table 2. However, we emphasize that the bulk of the results are due to inconsistencies, not errors.

**Table 1:** Naming convention statistics for selected ligands

**ligand code**	**ligand name**	**center atom**	***pro*-S**	***pro*-R**	**#**	**bias (%)**	**example PDB**
ACO	acetyl-coenzyme A	P1A	O1A	O2A	22	42%	1DM3
			O2A	O1A	30	58%	1B87
		P2A	O4A	O5A	25	48%	1B87
			O5A	O4A	27	52%	1DM3
ADP	adenosine-5'-diphosphate	PA	O1A	O2A	211	33%	1A6E
			O2A	O1A	419	67%	13PK
ATP	adenosine-5'-triphosphate	PA	O1A	O2A	103	30%	1B0U
			O2A	O1A	240	70%	1A0I
COA	coenzyme A	P1A	O1A	O2A	67	45%	1ACA
			O2A	O1A	81	55%	1CM0
		P2A	O4A	O5A	67	46%	1ESM
			O5A	O4A	78	54%	1ACA
CTP	cytidine-5'-triphosphate	PA	O1A	O2A	20	49%	1GQ9
			O2A	O1A	21	51%	1COZ
FAD	flavin-adenine dinucleotide	P	O1P	O2P	554	87%	1A8P
			O2P	O1P	81	13%	1B2R
		PA	O1A	O2A	290	46%	1AHV
			O2A	O1A	345	54%	1A8P
GTP	guanosine-5'-triphosphate	PA	O1A	O2A	35	36%	1CKM
			O2A	O1A	62	64%	1A8R
NAD	nicotinamide-adenine-dinucleotide	PA	O1A	O2A	144	27%	1A5Z
			O2A	O1A	388	73%	1A4Z
		PN	O1N	O2N	394	74%	1A4Z
			O2N	O1N	135	26%	1A7A
NAP	nadp nicotinamide-adenine-dinucleotide phosphate	PA	O1A	O2A	87	26%	1CIV
			O2A	O1A	247	74%	1A27
		PN	O1N	O2N	280	83%	1A27
			O2N	O1N	58	17%	1A80
TPP	thiamine diphosphate (i.e. vitamin B_1_)	PA	O1A	O2A	25	56%	1AY0
			O2A	O1A	20	44%	1B0P
UDP	uridine-5'-diphosphate	PA	O1A	O2A	80	79%	1BGU
			O2A	O1A	21	21%	1C3J

# = number of PDB files in which the given naming convention was observed.

### Examples of naming inconsistencies

Most of the atom naming inconsistencies mentioned in this paper relate to differences found between different files. However, there are a few cases in which naming inconsistencies can be found within a single file. One example is an X-ray crystal structure of alcohol dehydrogenase [PDB:2OHX] [16]. This structure contains two NAD molecules (see Figure 2). The prochiral center around phosphorus atom PN has consistent naming between the two molecules, however the prochiral center around phosphorus atom PA does not. In one case the *pro*-S and *pro*-R atoms are named "O1A" and "O2A", respectively, and in the other case, the names are "O2A" and "O1A", respectively.

**Figure 3:**
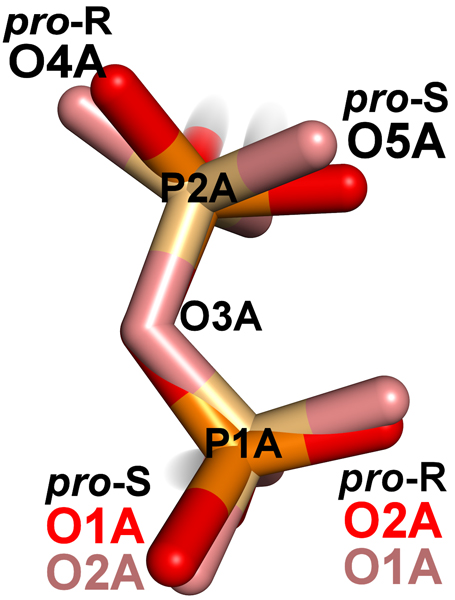
**Diphosphate of Coenzyme A from NMR structure **1NVL. The diphosphate region of coenzyme A of two models from 1NVL is shown. The diphosphate region of model 4 (light coloring) is superimposed on the same diphosphate region of model 2 (standard coloring). The diastereotopic names of prochiral center P2A have consistent names (O4A and O5A), but the *pro*-S and *pro*-R names for prochiral center P1A are not (O1A and O2A, respectively, for model 2, and O2A and O1A, respectively, for model 4).

Another example is an NMR structure of bovine acyl-coenzyme A binding protein (Figure 3) [PDB:1NVL]. This structure contained 20 NMR models, in which one phosphorus prochiral center was consistently named and the other was not. For the P1A center, models 1, 2, 5 and 18 have *pro*-S and *pro*-R atoms named "O1A" and "O2A", while the remaining 15 models have them named "O2A" and "O1A", respectively. Meanwhile, the *pro*-S and *pro*-R atoms at the P2A center are consistently named "O5A" and "O4A", respectively.

## Discussion

The inconsistent naming of atoms discussed in our paper is due largely to a lack of standardized names, *not *due to errors on the part of crystallographers or NMR researchers. There can be no errors where there are no rules.

A study of NAD(P) molecules by Carugo and Argos ignored the diastereotopic oxygen atoms for purposes of superimposing molecules because of naming inconsistencies [17]. Despite their use of atom-specific names for other atoms in the molecules, they only generally referred to diastereotopic oxygen atoms as "terminal oxygen atoms". That was eleven years ago and only involved a study of 32 protein structures. This was long before the recent remediation project of the PDB [18]. This project has done well to bring molecular and atomic naming conventions for PDB files into conformity with standards established by the International Union of Pure and Applied Chemistry (IUPAC) and the International Union of Biochemistry and Molecular Biology (IUBMB). However, IUPAC and IUBMB do not have standards for most diastereotopic atoms of small molecules.

There were no obvious overall trends in naming conventions with respect to the *pro*-R and *pro*-S atoms. This is likely due to the lack of naming standardization. However, trends are commonly seen among specific ligands (Table 1). One interesting observation is that the P prochiral center of FAD is highly biased in its naming convention (87% for one convention); however, the second center, PA has little bias (54% for one convention). Another observation is that NAD-like ligands tend to have naming conventions such that similar names (e.g. O1A and O1N) are seen on the same "side" of the molecule.

We suggest a general rule that names for *pro*-S atoms come alphanumerically before names for *pro*-R atoms. This is similar to the standard of using "OP1" for *pro*-S and "OP2" for *pro*-R in nucleic acids. The data indicates that there is no strong bias for this nor for its opposite convention among diphosphate containing ligands.

Regardless of what rules may become adopted, it is important to know to which atom a particular name refers. Establishing standard names and topologies that take prochirality into consideration will result in less confusion and more accuracy in studies involving small molecules. Until standards are adopted, individuals mining the data need to do their own standardization of the names. This naming can be enforced upfront, prior to the official release of data, or it can be enforced by individuals mining the data.

## Conclusion

Current naming conventions do not completely map unique names to unique diastereotopic atoms, resulting in possible confusion or error, or at least the need for researchers to impose their own naming standardization. We herein describe many cases of naming inconsistencies for small molecules containing diphosphate moieties. A future study will assess naming conventions of all atoms in the PDB, addressing more general issues of chirality and prochirality. The in-house software used in this study is available upon request.

## Methods

### Selection of small molecules for analysis

PDB files were selected from the January 7, 2008 "snapshot" of the Protein Data Bank. The search feature of the Protein Data Bank website  was used to select PDB codes for files containing ligands that had substructures matching the SMILES pattern "C~O~P(~O)(~O)~O~P(~O)(~O)~O". Here, "C" represents a carbon atom, "~" represents any bond, "O" represents oxygen, "P" represents phosphorus, and the parentheses indicate that the oxygen atoms inside them are bonded to the preceding phosphorus atom in the list, not to subsequent atoms in the list. This matches any ligand containing a (PO_4_)_2 _moiety, such as NAD, ATP, and Coenzyme A, resulting in a list of 4435 PDB codes.

Since the PDB files corresponding to these codes also included other ligands not meeting our criteria, we analyzed each of the small molecules within each PDB file and selected each one that met the following criteria: (1) It did not have the same residue name as an amino acid or nucleic acid, including names mapped to standard residue names via the "MODRES" record. (2) It had an entry in the Chemical Component Dictionary  from the PDB. (3) It had complete coordinates for the non-hydrogen atoms specified in the Chemical Component Dictionary. And (4), it had a diphosphate group attached to carbon, with the diphosphate group consisting of two phosphorus atoms, each covalently bonded to four oxygen atoms. We chose to analyze the prochiral phosphate centers adjacent to carbon atoms because of their abundance and because it allowed a simple and direct application of the CIP algorithm.

Atoms were considered to be covalently bonded if the distance between their centers was less than the sum of their covalent radii plus a cushion of 0.4 Å, following the custom of the Cambridge Structural Database (CSD) [19]. Covalent radii were obtained from the CSD website .

Also excluded were molecules that had alternate conformations that shared the same residue number. This guaranteed that any modeled alternate conformations would contain complete molecules. Those files containing diphosphates were further checked for phosphorus atoms having a prochiral configuration (see Determination of Prochiral Centers below). For those that did, the names of all four atoms attached to the prochiral center were recorded along with their relative stereochemical positions. Of the 4435 files originally selected, 4184 were found to have at least one ligand with a prochiral phosphate atom.

### Determination of prochiral centers

The CIP algorithm [20,21] for assigning priorities to atoms within a molecule was implemented using in-house software. CIP priorities were calculated for all four atoms connected to a phosphorus atom. Following the CIP-algorithm, the oxygen atom attached to two phosphorus atoms always had the highest priority and the oxygen atom attached to carbon always had the second highest priority. The two remaining oxygen atoms were not bonded to any other atom besides the phosphorus atom.

If each atom had a distinct priority, then the phosphorus is chiral and the determinant algorithm of Cieplak and Wisniewski[22] could be used to calculate whether the configuration is R or S as shown in Equation (1):

(1) $$\left|\begin{array}{cccc} X_{A} & Y_{A} & Z_{A} & 1\\ X_{B} & Y_{B} & Z_{B} & 1\\ X_{C} & Y_{C} & Z_{C} & 1\\ X_{D} & Y_{D} & Z_{D} & 1 \end{array}\right|=m$$

X_N_, Y_N_, and Z_N _are the x, y, and z components of the coordinates for group N. The subscripted letters A, B, C, and D represent the highest, second highest, third highest, and lowest priority atoms, respectively (see Figure 1). *m *is the result of calculating the determinant. It is negative for the R configuration and positive for the S configuration. If it is evaluated to be zero, then the atoms are all in the same plane [22], which should never be the case for tetrahedrally arranged molecules such as phosphates. For understanding the mathematics behind this equation and how it captures the handedness of four three-dimensional coordinates, we refer the reader to the work of Cieplak and Wisniewski [22].

If two of the atoms attached to the phosphorus atom have identical priorities, then they are diastereotopic and the phosphorus is prochiral. In the case of diphosphate-containing molecules, the diastereotopic atoms are only bonded to phosphorus and therefore have the lowest priority (see Figure 2). We will call the atoms attached to the phosphorus atom A, B, C, and C', where A and B have the highest and second highest priority, respectively, while C and C' tie for the lowest priority. In this case, Equation (1) can be adapted to determine whether C is the *pro*-S or *pro*-R atom and, concomitantly, whether C' is the *pro*-R or *pro*-S atom. By definition, a diastereotopic atom being *pro*-S (or *pro*-R) means that, if it were replaced by a group with higher priority than any other substituent, then the prochiral center would become chiral with an S (or R) configuration. Therefore, we treat C as if it had the highest priority and then calculate the resulting configuration. If the calculated configuration is S, then C is *pro*-S; if it is R, then C is *pro*-R. To do this computationally, we artificially raise the priority of C to be the highest (i.e. higher than A) changing Equation (1) to the following:

(2) $$\left|\begin{array}{cccc} X_{C} & Y_{C} & Z_{C} & 1\\ X_{A} & Y_{A} & Z_{A} & 1\\ X_{B} & Y_{B} & Z_{B} & 1\\ X_{C^{\prime }} & Y_{C^{\prime }} & Z_{C^{\prime }} & 1 \end{array}\right|=m$$

If m is positive, then C is the *pro*-S atom and, concomitantly, C' is the *pro*-R atom (Figure 2). If m is negative, then C is the *pro*-R atom and C' is the *pro*-S atom.

### Third-party software used

COOT [23] was used for visualizing PDB files, which was especially useful during the development of our software. As needed, the SSM [24] module of COOT was also used for superposition of molecules. Pymol was used for viewing NMR models as well as generating depictions of molecular structures for figures [25].

## Competing interests

The authors declare that they have no competing interests.

## Authors' contributions

CAB participated in the design of the study, developed the in-house software, carried out the atom name analysis, and drafted the manuscript. DX participated in the design and coordination of the study, and helped draft the manuscript. Both authors read and approved the final manuscript.

## Supplementary Material

Additional file 1 **Supplemental Table 1**. Contains Table 1 from this document with about four additional pages of examples of naming convention statistics for selected ligands. Click here for file

Additional file 2 **Supplemental Table 2**. Contains all of the calculated results, including those for prochiral centers that appear only once in the PDB. Click here for file

Additional file 3 **Explanation of Supplemental Table 2**. Contains an explanation of the columns in Supplemental Table 2. Click here for file
