# Supplementary material for: Wanted: unique names for unique atom positions. PDB-wide analysis of diastereotopic atom names of small molecules containing diphosphate
Source: BMC Bioinformatics. 2008 Aug 12;9(Suppl 9):S16. doi: 10.1186/1471-2105-9-S9-S16 (PMC2537567; doi:10.1186/1471-2105-9-S9-S16)
Supplement: Additional file 1 — Supplemental Table 1. Contains Table 1 from this document with about four additional pages of examples of naming convention statistics for selected ligands. [file 1471-2105-9-S9-S16-S1.doc]

| Supplemental Table 1. Naming convention statistics for selected ligands | | | | | | | |
| --- | --- | --- | --- | --- | --- | --- | --- |
| ligand code | ligand name | center  atom | pro-S | pro-R | # | bias  (%) | example PDB |
| 2MD | bis(molybdopterin guanine dinucleotide)molybdenum  cofactor | PB | O1B | O2B | 3 | 75% | 1H0H |
|  |  |  | O2B | O1B | 1 | 25% | 1TMO |
| 4TA | p1-(5'-adenosyl)p4-(5'-(2'-deoxy-thymidyl))tetra­phosphate | PD | O1D | O2D | 1 | 50% | 2ORW |
|  |  |  | O2D | O1D | 1 | 50% | 1P73 |
| ACO | acetyl-coenzyme A | P1A | O1A | O2A | 22 | 42% | 1DM3 |
|  |  |  | O2A | O1A | 30 | 58% | 1B87 |
|  |  | P2A | O4A | O5A | 25 | 48% | 1B87 |
|  |  |  | O5A | O4A | 27 | 52% | 1DM3 |
| ADJ | nicotinamide-adenine-dinucleotide-adenylate intermediate | PB | O1B | O2B | 1 | 50% | 1KQP |
|  |  |  | O2B | O1B | 1 | 50% | 1KQP |
| ADP | adenosine-5'-diphosphate | PA | O1A | O2A | 211 | 33% | 1A6E |
|  |  |  | O2A | O1A | 419 | 67% | 13PK |
| ADQ | adenosine-5'-diphosphate-glucose | PB | O1B | O2B | 1 | 25% | 1EQ2 |
|  |  |  | O2B | O1B | 3 | 75% | 1EQ2 |
| AGS | phosphothiophosphoric acid-adenylate ester | PA | O1A | O2A | 3 | 30% | 1XPO |
|  |  |  | O2A | O1A | 7 | 70% | 1KJJ |
| AP0 | acetyl pyridine adenine dinucleotide, reduced | PA | O1A | O2A | 1 | 50% | 2AA3 |
|  |  |  | O2A | O1A | 1 | 50% | 2A94 |
| AP5 | bis(adenosine)-5'-pentaphosphate | PA | O1A | O2A | 1 | 6% | 1Z83 |
|  |  |  | O2A | O1A | 15 | 94% | 1AKE |
|  |  | PE | O1E | O2E | 2 | 13% | 1Z83 |
|  |  |  | O2E | O1E | 14 | 88% | 1AKE |
| APR | adenosine-5-diphosphoribose | PA | O1A | O2A | 5 | 17% | 1S7G |
|  |  |  | O2A | O1A | 25 | 83% | 1G9Q |
|  |  | PB | O1B | O2B | 28 | 93% | 1G9Q |
|  |  |  | O2B | O1B | 2 | 7% | 1IHY |
| ATG | phosphothiophosphoric acid-adenylate ester | PA | O1A | O2A | 1 | 10% | 1H7U |
|  |  |  | O2A | O1A | 9 | 90% | 1BIF |
| ATP | adenosine-5'-triphosphate | PA | O1A | O2A | 103 | 30% | 1B0U |
|  |  |  | O2A | O1A | 240 | 70% | 1A0I |
| ATR | 2'-monophospho­adenosine-5'-diphosphate | PA | O1A | O2A | 2 | 50% | 1AFL |
|  |  |  | O2A | O1A | 2 | 50% | 1P77 |
| CAA | acetoacetyl-coenzyme A | P1A | O1A | O2A | 15 | 94% | 1BUC |
|  |  |  | O2A | O1A | 1 | 6% | 2UZF |
|  |  | P2A | O4A | O5A | 1 | 6% | 2UZF |
|  |  |  | O5A | O4A | 15 | 94% | 1BUC |
| CAO | oxidized coenzyme A | P1A | O1A | O2A | 1 | 17% | 1EAD |
|  |  |  | O2A | O1A | 5 | 83% | 1T3Z |
|  |  | P2A | O4A | O5A | 5 | 83% | 1T3Z |
|  |  |  | O5A | O4A | 1 | 17% | 1EAD |
| CDM | 4-diphosphocytidyl-2-c-methyl-d-erythritol | PB | O1B | O2B | 3 | 75% | 1INI |
|  |  |  | O2B | O1B | 1 | 25% | 1U40 |
| CDP | cytidine-5'-diphosphate | PA | O1A | O2A | 10 | 91% | 1FFU |
|  |  |  | O2A | O1A | 1 | 9% | 2AZ3 |
| CMC | carboxymethyl coenzyme A* | P1A | O1A | O2A | 1 | 33% | 5CTS |
|  |  |  | O2A | O1A | 2 | 67% | 1CSC |
| COA | coenzyme A | P1A | O1A | O2A | 67 | 45% | 1ACA |
|  |  |  | O2A | O1A | 81 | 55% | 1CM0 |
|  |  | P2A | O4A | O5A | 67 | 46% | 1ESM |
|  |  |  | O5A | O4A | 78 | 54% | 1ACA |
| COD | dephospho coenzyme A | P20 | O21 | O22 | 1 | 50% | 1B6T |
|  |  |  | O22 | O21 | 1 | 50% | 2GRJ |
|  |  | P24 | O25 | O26 | 1 | 50% | 2GRJ |
|  |  |  | O26 | O25 | 1 | 50% | 1B6T |
| COS | coenzyme A persulfide | P1A | O1A | O2A | 1 | 33% | 1IVH |
|  |  |  | O2A | O1A | 2 | 67% | 2JIF |
|  |  | P2A | O4A | O5A | 2 | 67% | 2JIF |
|  |  |  | O5A | O4A | 1 | 33% | 1IVH |
| COT | coa-s-acetyl tryptamine | PA | O15 | O16 | 1 | 33% | 1L0C |
|  |  |  | O16 | O15 | 2 | 67% | 1CJW |
| COZ | coenzyme A* | P2A | O4A | O5A | 1 | 50% | 2G2Z |
|  |  |  | O5A | O4A | 1 | 50% | 2C6X |
| CTP | cytidine-5'-triphosphate | PA | O1A | O2A | 20 | 49% | 1GQ9 |
|  |  |  | O2A | O1A | 21 | 51% | 1COZ |
| D3T | 2',3'-dideoxy-thymidine-5'-triphosphate | PA | O1A | O2A | 2 | 50% | 1TK8 |
|  |  |  | O2A | O1A | 2 | 50% | 1XSN |
| DAD | 2',3'-dideoxyadenosine-5'-triphosphate | PA | O1A | O2A | 6 | 55% | 1CJT |
|  |  |  | O2A | O1A | 5 | 45% | 1KEJ |
| DAU | 2'deoxy-thymidine-5'-diphospho-alpha-d-glucose | P | O1P | O2P | 2 | 33% | 1H5T |
|  |  |  | O2P | O1P | 4 | 67% | 1G1L |
|  |  | P2 | O3P | O4P | 4 | 67% | 1G1L |
|  |  |  | O4P | O3P | 2 | 33% | 1H5T |
| DCA | desulfo-coenzyme A | P1A | O1A | O2A | 1 | 20% | 2OI7 |
|  |  |  | O2A | O1A | 4 | 80% | 1E1C |
| DCP | 2'-deoxycytidine-5'-triphosphate | PA | O1A | O2A | 10 | 43% | 1LV5 |
|  |  |  | O2A | O1A | 13 | 57% | 1H78 |
| DCT | 2',3'-dideoxycytidine 5'-triphosphate | PA | O1A | O2A | 6 | 40% | 1T8E |
|  |  |  | O2A | O1A | 9 | 60% | 1BPY |
| DGT | 2'-deoxyguanosine-5'-triphosphate | PA | O1A | O2A | 13 | 59% | 1XJJ |
|  |  |  | O2A | O1A | 9 | 41% | 1HK8 |
| DMA | dimethylallyl diphosphate | PA | O1A | O2A | 2 | 67% | 1UBY |
|  |  |  | O2A | O1A | 1 | 33% | 1YHL |
| DND | nicotinic acid adenine dinucleotide | PA | O13 | O14 | 2 | 22% | 1XQD |
|  |  |  | O14 | O13 | 7 | 78% | 1EE1 |
|  |  | PN | O11 | O12 | 4 | 44% | 1KAQ |
|  |  |  | O12 | O11 | 5 | 56% | 1EE1 |
| DTP | 2'-deoxyadenosine 5'-triphosphate | PA | O1A | O2A | 8 | 38% | 1S0M |
|  |  |  | O2A | O1A | 13 | 62% | 1CR2 |
| DUD | deoxyuridine-5'-diphosphate | PA | O1A | O2A | 6 | 75% | 1DUD |
|  |  |  | O2A | O1A | 2 | 25% | 1DUC |
| DUT | deoxyuridine-5'-triphosphate | PA | O1A | O2A | 9 | 90% | 1EXC |
|  |  |  | O2A | O1A | 1 | 10% | 2NOM |
| EIP | 4-hydroxy-3-methyl butyl diphosphate | P8 | O6 | O7 | 3 | 75% | 1NFZ |
|  |  |  | O7 | O6 | 1 | 25% | 1OW2 |
| EPU | uridine-diphosphate-2(n-acetylglucosaminyl) butyric acid | PA | O1A | O2A | 5 | 83% | 1P31 |
|  |  |  | O2A | O1A | 1 | 17% | 1RYW |
| FAD | flavin-adenine dinucleotide | P | O1P | O2P | 554 | 87% | 1A8P |
|  |  |  | O2P | O1P | 81 | 13% | 1B2R |
|  |  | PA | O1A | O2A | 290 | 46% | 1AHV |
|  |  |  | O2A | O1A | 345 | 54% | 1A8P |
| FDA | dihydroflavine-adenine dinucleotide | P | O1P | O2P | 2 | 50% | 1FNC |
|  |  |  | O2P | O1P | 2 | 50% | 2ARD |
|  |  | PA | O1A | O2A | 1 | 25% | 2ARD |
|  |  |  | O2A | O1A | 3 | 75% | 1FNC |
| FPP | farnesyl diphosphate | PA | O1A | O2A | 17 | 89% | 1FPP |
|  |  |  | O2A | O1A | 2 | 11% | 1O5M |
| G3A | guanosine-p3-adenosine-5',5'-triphosphate | PG | O1G | O2G | 1 | 50% | 2P3L |
|  |  |  | O2G | O1G | 1 | 50% | 2PXC |
| G4P | guanosine-5',3'-tetraphosphate | PA | O1A | O2A | 1 | 33% | 2J4R |
|  |  |  | O2A | O1A | 2 | 67% | 1LNZ |
|  |  | PC | O1C | O2C | 1 | 33% | 2J4R |
|  |  |  | O2C | O1C | 2 | 67% | 1LNZ |
| GDC | guanosine-5'-diphosphate-beta-l-galactose | P | O1P | O3P | 1 | 50% | 2C59 |
|  |  |  | O3P | O1P | 1 | 50% | 2C5A |
| GDD | guanosine-5'-diphosphate-alpha-d-mannose | PA | O1A | O2A | 4 | 80% | 2C5E |
|  |  |  | O2A | O1A | 1 | 20% | 2C59 |
| GDP | guanosine-5'-diphosphate | PA | O1A | O2A | 99 | 32% | 1A4R |
|  |  |  | O2A | O1A | 211 | 68% | 1A2K |
| GDU | galactose-uridine-5'-diphosphate | PA | O1A | O2A | 7 | 88% | 1GUP |
|  |  |  | O2A | O1A | 1 | 13% | 1I3L |
|  |  | PB | O1B | O2B | 4 | 50% | 1I3L |
|  |  |  | O2B | O1B | 4 | 50% | 1GUP |
| GP3 | diguanosine-5'-triphosphate | PG | O1G | O2G | 1 | 50% | 1CKO |
|  |  |  | O2G | O1G | 1 | 50% | 2JHA |
| GPP | geranyl diphosphate | PA | O1A | O2A | 8 | 89% | 1UBW |
|  |  |  | O2A | O1A | 1 | 11% | 1ZCW |
| GRG | geranylgeranyl diphosphate | PA | O1A | O2A | 5 | 83% | 1N4P |
|  |  |  | O2A | O1A | 1 | 17% | 2J1P |
| GTA | p1-7-methylguanosine-p3-adenosine-5',5'-triphosphate | P1 | O11 | O12 | 2 | 50% | 1IPB |
|  |  |  | O12 | O11 | 2 | 50% | 1ST4 |
|  |  | P3 | O31 | O32 | 1 | 20% | 1ST4 |
|  |  |  | O32 | O31 | 4 | 80% | 1IPB |
| GTG | mrna cap analog n7-methyl gpppg | PA | O1A | O2A | 3 | 75% | 1MWH |
|  |  |  | O2A | O1A | 1 | 25% | 1ST0 |
| GTP | guanosine-5'-triphosphate | PA | O1A | O2A | 35 | 36% | 1CKM |
|  |  |  | O2A | O1A | 62 | 64% | 1A8R |
| GUD | glucose-uridine-c1,5'-diphosphate | PA | O1A | O2A | 3 | 75% | 1GUQ |
|  |  |  | O2A | O1A | 1 | 25% | 2PA4 |
|  |  | PB | O1B | O2B | 1 | 25% | 1O23 |
|  |  |  | O2B | O1B | 3 | 75% | 1GUQ |
| HMG | 3-hydroxy-3-methylglutaryl-coenzyme A | P2A | O4A | O5A | 2 | 50% | 1XPK |
|  |  |  | O5A | O4A | 2 | 50% | 1DQ9 |
| HTL | 2-acetyl-thiamine diphosphate | P1 | O12 | O13 | 3 | 75% | 1KEK |
|  |  |  | O13 | O12 | 1 | 25% | 2C3Y |
| HXC | hexanoyl-coenzyme A | P1 | O11 | O12 | 1 | 33% | 1W6U |
|  |  |  | O12 | O11 | 2 | 67% | 1MJ3 |
| IPE | 3-methylbut-3-enyl trihydrogen diphosphate | PA | O1A | O2A | 7 | 88% | 1X07 |
|  |  |  | O2A | O1A | 1 | 13% | 2F8Z |
| ITT | inosine 5'-triphosphate | PA | O1A | O2A | 1 | 33% | 2J4E |
|  |  |  | O2A | O1A | 2 | 67% | 2DVO |
| M7G | 7n-methyl-8-hydroguanosine-5'-diphosphate | PA | O1A | O2A | 3 | 30% | 1RF8 |
|  |  |  | O2A | O1A | 7 | 70% | 1AP8 |
| MCN | pterin cytosine dinucleotide | PA | O1A | O2A | 7 | 88% | 1N5W |
|  |  |  | O2A | O1A | 1 | 13% | 1DGJ |
|  |  | PB | O1B | O2B | 7 | 88% | 1N5W |
|  |  |  | O2B | O1B | 1 | 13% | 1DGJ |
| MD1 | phosphoric acid 4-(2-amino-4-oxo-3,4,5,6,-tetrahydro-pteridin-6-yl)-2-hydroxy-3,4-dimercapto-but-3-en-yl ester guanylate ester | PA | O1A | O2A | 5 | 83% | 1Q16 |
|  |  |  | O2A | O1A | 1 | 17% | 2IVF |
| MGD | 2-amino-5,6-dimercapto-7-methyl-3,7,8a,9-tetrahydro-8-oxa-1,3,9,10-tetraaza-anthracen-4-one guanosine dinucleotide* | PA | O1A | O2A | 13 | 48% | 1H0H |
|  |  |  | O2A | O1A | 14 | 52% | 1AA6 |
|  |  | PB | O1B | O2B | 12 | 57% | 1AA6 |
|  |  |  | O2B | O1B | 9 | 43% | 1OGY |
| MLC | malonyl-coenzyme A | P1 | O11 | O12 | 1 | 33% | 2E1T |
|  |  |  | O12 | O11 | 2 | 67% | 1CML |
|  |  | P2 | O21 | O22 | 2 | 67% | 1CML |
|  |  |  | O22 | O21 | 1 | 33% | 2E1T |
| MYA | tetradecanoyl-coa | P1A | O1A | O2A | 5 | 83% | 1IIC |
|  |  |  | O2A | O1A | 1 | 17% | 2CB8 |
|  |  | P2A | O4A | O5A | 4 | 67% | 1IIC |
|  |  |  | O5A | O4A | 2 | 33% | 1IYK |
| NAD | nicotinamide-adenine-dinucleotide | PA | O1A | O2A | 144 | 27% | 1A5Z |
|  |  |  | O2A | O1A | 388 | 73% | 1A4Z |
|  |  | PN | O1N | O2N | 394 | 74% | 1A4Z |
|  |  |  | O2N | O1N | 135 | 26% | 1A7A |
| NAI | 1,4-dihydronicotinamide adenine dinucleotide | PA | O1A | O2A | 5 | 13% | 1I0Z |
|  |  |  | O2A | O1A | 35 | 88% | 1DLJ |
|  |  | PN | O1N | O2N | 32 | 80% | 1DLJ |
|  |  |  | O2N | O1N | 8 | 20% | 1I0Z |
| NAP | nadp nicotinamide-adenine-dinucleotide phosphate | PA | O1A | O2A | 87 | 26% | 1CIV |
|  |  |  | O2A | O1A | 247 | 74% | 1A27 |
|  |  | PN | O1N | O2N | 280 | 83% | 1A27 |
|  |  |  | O2N | O1N | 58 | 17% | 1A80 |
| NDP | nadph dihydro-nicotinamide-adenine-dinucleotide phosphate | PA | O1A | O2A | 80 | 44% | 1A4I |
|  |  |  | O2A | O1A | 103 | 56% | 1ABN |
|  |  | PN | O1N | O2N | 131 | 70% | 1A4I |
|  |  |  | O2N | O1N | 57 | 30% | 1AI9 |
| P22 | ethyl dihydrogen diphosphate | PA | O1A | O2A | 6 | 75% | 1T9B |
|  |  |  | O2A | O1A | 2 | 25% | 1YI0 |
| PAP | 3'-phosphate-adenosine-5'-diphosphate | PA | O1A | O2A | 3 | 75% | 1AFK |
|  |  |  | O2A | O1A | 1 | 25% | 1M4G |
| PCP | 1-alpha-pyrophosphoryl-2-alpha,3-alpha-dihydroxy-4-beta-cyclo­pentane-methanol-5-phosphate | PA | O1A | O2A | 2 | 33% | 1ECC |
|  |  |  | O2A | O1A | 4 | 67% | 1A95 |
| PGD | 2-amino-5,6-dimercapto-7-methyl-3,7,8a,9-tetrahydro-8-oxa-1,3,9,10-tetraaza-anthracen-4-one guanosine dinucleotide* | PA | O1A | O2A | 10 | 50% | 1DMR |
|  |  |  | O2A | O1A | 10 | 50% | 1DMR |
|  |  | PB | O1B | O2B | 8 | 80% | 1DMR |
|  |  |  | O2B | O1B | 2 | 20% | 2DMR |
| PRP | alpha-phosphoribosylpyro­phosphoric acid | PA | O1A | O2A | 18 | 95% | 1D6N |
|  |  |  | O2A | O1A | 1 | 5% | 2E5C |
| SAP | adenosine-5'-diphosphate monothiophosphate | PA | O1A | O2A | 3 | 23% | 1GJV |
|  |  |  | O2A | O1A | 10 | 77% | 1AUX |
| SND | thionicotinamide-adenine-dinucleotide | PN | O1N | O2N | 1 | 50% | 1PTJ |
|  |  |  | O2N | O1N | 1 | 50% | 1IHX |
| T5A | p1-(5'-adenosyl)p5-(5'-thymidyl)pentaphosphate | PE | O1E | O2E | 1 | 33% | 1P7C |
|  |  |  | O2E | O1E | 2 | 67% | 1MRN |
| TDP | thiamin diphosphate* | P1 | O12 | O13 | 16 | 52% | 1DTW |
|  |  |  | O13 | O12 | 15 | 48% | 1OLS |
| TPP | thiamine diphosphate* | PA | O1A | O2A | 25 | 56% | 1AY0 |
|  |  |  | O2A | O1A | 20 | 44% | 1B0P |
| TTP | thymidine-5'-triphosphate | PA | O1A | O2A | 8 | 25% | 1G2V |
|  |  |  | O2A | O1A | 24 | 75% | 1CR1 |
| TYD | thymidine-5'- diphosphate | PA | O1A | O2A | 10 | 42% | 1E2G |
|  |  |  | O2A | O1A | 14 | 58% | 1CR4 |
| UD1 | uridine-diphosphate-n-acetylglucosamine | PA | O1A | O2A | 9 | 35% | 1G97 |
|  |  |  | O2A | O1A | 17 | 65% | 1FOA |
|  |  | PB | O1B | O2B | 20 | 77% | 1FOA |
|  |  |  | O2B | O1B | 6 | 23% | 1HZJ |
| UD2 | uridine-diphosphate-n-acetylgalactosamine | PA | O1A | O2A | 5 | 63% | 1I3N |
|  |  |  | O2A | O1A | 3 | 38% | 1JV3 |
|  |  | PB | O1B | O2B | 5 | 56% | 1JV3 |
|  |  |  | O2B | O1B | 4 | 44% | 1I3N |
| UDH | 6-aminohexyl-uridine-c1,5'-diphosphate | PB | O1B | O3B | 1 | 17% | 1TW5 |
|  |  |  | O3B | O1B | 5 | 83% | 2AE7 |
| UDP | uridine-5'-diphosphate | PA | O1A | O2A | 80 | 79% | 1BGU |
|  |  |  | O2A | O1A | 21 | 21% | 1C3J |
| UPG | uridine-5'-diphosphate-glucose | PA | O1A | O2A | 26 | 79% | 1A9Y |
|  |  |  | O2A | O1A | 7 | 21% | 1EK6 |
|  |  | PB | O1B | O2B | 17 | 52% | 1EK6 |
|  |  |  | O2B | O1B | 16 | 48% | 1A9Y |
| UTP | uridine 5'-triphosphate | PA | O1A | O2A | 12 | 63% | 1GX6 |
|  |  |  | O2A | O1A | 7 | 37% | 1NB6 |
| ZID | isonicotinic-acetyl-nicotinamide-adenine dinucleotide | PA | O1A | O2A | 1 | 14% | 2NQ8 |
|  |  |  | O2A | O1A | 6 | 86% | 1ZID |
|  |  | PN | O1N | O2N | 6 | 86% | 1ZID |
|  |  |  | O2N | O1N | 1 | 14% | 2NQ8 |

*Some of these ligands have identical names, but there are actually differences in chirality or differences in hydrogen atoms.

# = number of PDB files in which the given naming convention was observed. Multiple naming conventions can be observed within the same file. For example, for ligand PGD and center PA, there are 10 PDB files containing one convention as well as 10 containing a different convention. Nonetheless, the example PDB listed for both conventions is 1DMR because, alphanumerically, its code was the first among those representing each convention.
