# Supplementary material for: Wanted: unique names for unique atom positions. PDB-wide analysis of diastereotopic atom names of small molecules containing diphosphate
Source: BMC Bioinformatics. 2008 Aug 12;9(Suppl 9):S16. doi: 10.1186/1471-2105-9-S9-S16 (PMC2537567; doi:10.1186/1471-2105-9-S9-S16)
Supplement: Additional file 3 — Explanation of Supplemental Table 2. Contains an explanation of the columns in Supplemental Table 2. [file 1471-2105-9-S9-S16-S3.doc]

**Explanation of column names and content for All_Results.xls**

**pdb code**

Four letter PDB accession code. To get more information about a particular PDB file, clik on this code, which is hyperlinked. For example, the code "13PK" is hyperlinked to <http://www.rcsb.org/pdb/explore/explore.do?structureId=13PK>.

**model**

For files containing a single model, this number is 0. For files containing more than one model (e.g. NMR models), this number indicates which model the contents of the record (line) belong to.

**ligand code**

Unique code for a particular small molecule that can be found in the Protein Data Bank. To get information about a particular ligand, simply click on the code, which is hyperlinked. For example, the code "ADP" is hyperlinked to <http://remediation.wwpdb.org/pyapps/ldHandler.py?query=ADP>.

**prochiral center**

Name of a prochiral atom from the indicated ligand from the indicated PDB file. It is the center of the prochiral phosphate. The atoms attached to it are listed in the columns "Highest priority", "Next Highest Priority", "pro-S", and "pro-R".

**chain ID**

Name of the chain ID of the molecule analyzed.

**residue number**

Residue number of the molecule analyzed.

**Highest priority**

Name of the highest priority atom attached to the prochiral center. This is the oxygen atom that is attached to two phosphorus atoms, the phosphorus atom listed in the prochiral center column and another phosphorus atom of the adjacent phosphate group.

**Next-highest priority**

Name of the next-highest priority atom attached to the phosphorus atom listed in the prochiral center column.

***pro*-S**

Name of the *pro*-S atom attached to the phosphorus atom listed in the prochiral center column.

***pro*-R**

Name of the *pro*-R atom attached to the phosphorus atom listed in the prochiral center column.

**An explanation about atom names.**

As in the PDB file, atom names in this file contain four characters, the first two characters containing the atomic symbol and the last two characters indicating position within a molecule. Since all of the atoms indicated in this (i.e. phosphorus and oxygen) have single character atomic symbols, the first character in each name is a space. Many atom names also end in a space.

**Notes for users who wish to save this file in another format.**

Potential problem for formats that use quotes: Many atom names in this file contain a single quote as part of the name.

Potential problem for comma-delimited formats: A few atom names include commas (e.g. [DAU](http://remediation.wwpdb.org/pyapps/ldHandler.py?query=DAU)). This is not an error, as it is specified in the Chemical Component Dictionary (<http://remediation.wwpdb.org/downloads/Components-rel-alt.cif>).
